# Argon reduces the pulmonary vascular tone in rats and humans by GABA-receptor activation

**DOI:** 10.1038/s41598-018-38267-y

**Published:** 2019-02-13

**Authors:** Said Suleiman, Sergej Klassen, Ira Katz, Galina Balakirski, Julia Krabbe, Saskia von Stillfried, Svetlana Kintsler, Till Braunschweig, Aaron Babendreyer, Jan Spillner, Sebastian Kalverkamp, Thomas Schröder, Manfred Moeller, Mark Coburn, Stefan Uhlig, Christian Martin, Annette D. Rieg

**Affiliations:** 1 Institute of Pharmacology and Toxicology, Medical Faculty RWTH-Aachen, 52074 Aachen, Germany; 2 Medical Research & Development, Air Liquide Santé Internationale, Centre de Recherche Paris-Saclay, 78354 Jouy-en-Josas, France; 3 Institute of Pathology, Medical Faculty RWTH Aachen, 52074 Aachen, Germany; 4 Department of Cardiac and Thoracic Surgery, Medical Faculty RWTH Aachen, 52074 Aachen, Germany; 5 Department of Surgery, Luisenhospital Aachen, 52064 Aachen, Germany; 6 Institute for Occupational, Social and Environmental Medicine, Medical Faculty RWTH Aachen, 52074 Aachen, Germany; 7 Department of Anaesthesiology, Medical Faculty RWTH Aachen, 52074 Aachen, Germany

## Abstract

Argon exerts neuroprotection. Thus, it might improve patients’ neurological outcome after cerebral disorders or cardiopulmonary resuscitation. However, limited data are available concerning its effect on pulmonary vessel and airways. We used rat isolated perfused lungs (IPL) and precision-cut lung slices (PCLS) of rats and humans to assess this topic. IPL: Airway and perfusion parameters, oedema formation and the pulmonary capillary pressure (P_cap_) were measured and the precapillary and postcapillary resistance (R_post_) was calculated. In IPLs and PCLS, the pulmonary vessel tone was enhanced with ET-1 or remained unchanged. IPLs were ventilated and PCLS were gassed with argon-mixture or room-air. IPL: Argon reduced the ET-1-induced increase of P_cap_, R_post_ and oedema formation (p < 0.05). PCLS (rat): Argon relaxed naïve pulmonary arteries (PAs) (p < 0.05). PCLS (rat/human): Argon attenuated the ET-1-induced contraction in PAs (p < 0.05). Inhibition of GABA_B_-receptors abolished argon-induced relaxation (p < 0.05) in naïve or ET-1-pre-contracted PAs; whereas inhibition of GABA_A_-receptors only affected ET-1-pre-contracted PAs (p < 0.01). GABA_A/B_-receptor agonists attenuated ET-1-induced contraction in PAs and baclofen (GABA_B_-agonist) even in pulmonary veins (p < 0.001). PLCS (rat): Argon did not affect the airways. Finally, argon decreases the pulmonary vessel tone by activation of GABA-receptors. Hence, argon might be applicable in patients with pulmonary hypertension and right ventricular failure.

## Introduction

Noble gases were considered to be inert due to their filled outer electron shell: Meanwhile it is recognised that they exert physiological effects by van der Waals forces^1,2^. The protective effects of argon and xenon on cellular integrity have been shown for numerous conditions being at high risk for organ dysfunction or poor cerebral outcome, e.g. cardiac surgery^3–6^, cardiac resuscitation^7–10^, transplantation^11^ or neurological disorders^12–18^. The mechanisms beyond xenon-induced neuroprotection comprise NMDA-antagonism and activation of two-pore potassium channels (TREK-1) or K_ATP_-channels^1^. Referring the neuroprotective effects of argon, several mechanisms are discussed; e.g. activation of ERK1/2 and PI3K-AKT^3,19,20^, stimulation of TLR2/4^17,21,22^ and up-regulation of the anti-apoptotic gene Bcl-2^18^. Recently, a common mechanism of argon and xenon has been identified. Both noble gases desensitise acid-sensing ion channels which was shown to be neuroprotective in mouse models of ischaemic stroke^23^. Regarding the anaesthetic effect of argon under hyperbaric conditions, GABA_A_-receptors appear to be involved^24^.

The use of xenon is limited due to its rarity of 0.09 ppm in the atmosphere; in contrast argon is abundant at 0.93%^25^. The clinical application of argon appears to be more conceivable. The fact that it is non-anaesthetic at normobaric pressure might be even advantageous, as patients requiring neuroprotection are rather harmed from additional sedation. Concerning the neuroprotective effects of argon, only experimental data are available thus far. Yet, one study in humans confirmed that short-term exposure to argon does not affect cerebral circulation or metabolism^26^.

Usually, neuroprotection is warranted in patients suffering primary neurological disorder (traumatic brain injury, cerebral ischaemia and bleeding) or secondary cerebral ischaemia due to cardiac arrest and cardiac surgery. These patients are often affected by cardiovascular and pulmonary disorders; e.g. left heart disease (LHD), right ventricular (RV) failure, pulmonary hypertension (PH), chronical asthma or chronic obstructive lung disease. Hence, the effects of inhaled argon on airway or pulmonary haemodynamic parameters should be considered. Currently, clinical studies addressing this topic are lacking and experimental trials are rare^27,28^. One trial in newborn pigs assessed the systemic vascular effects of argon and showed that ventilation with argon (80%) does not affect heart rate or mean arterial blood pressure^12,29^. Further, Martens *et al*.^27,28^ showed that ventilation with argon (70% or 79%) does not affect total pulmonary vascular resistance (PVR). However segmental PVR, expressed as precapillary (R_pre_) and postcapillary resistance (R_post_) gives much more evidence about the pulmonary arterial and venous bed. This topic is even more relevant, as pulmonary arteries (PAs) and veins (PVs) react quite differently^30–32^ and PH due to LHD primarily affects PVs^33,34^.

We studied the effects of argon on R_pre_ and R_post_ in isolated perfused lungs (IPL)^35^ which allows for the measurement of the capillary pressure (P_cap_) and the calculation of R_pre_ and R_post_. The direct effects of argon and the role of GABA were studied in precision-cut lung slices (PCLS) from rats or humans^31,36^. PCLS are viable for 72 hours and enable the real time evaluation of the tone of PAs, PVs and airways^31,36,37^.

## Results

We evaluated the effects of argon on pulmonary haemodynamic and airway parameters using rat isolated perfused lungs (IPL) and precision-cut lung slices (PCLS) of rats and humans. In both models, healthy lungs with or without ET-1 pre-treatment were studied. In addition, we addressed the role of GABA-receptor activation within argon-induced relaxation in naïve or ET-1 pre-contracted rat PCLS.

### The effects of argon on pulmonary haemodynamics and airway parameters in the IPL

**Figure 1:**
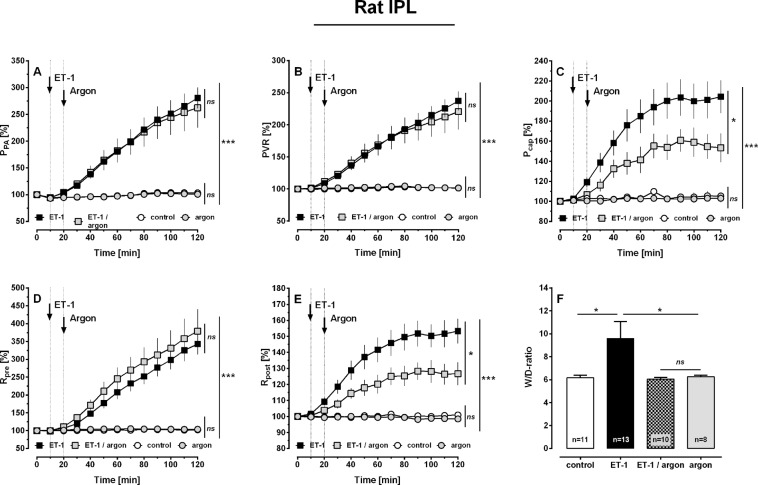
Influence of argon on pulmonary haemodynamics in the IPL of the rat. (**A**) Pulmonary arterial pressure (P_PA_); (**B**) PVR; (**C**) Pulmonary capillary pressure (P_cap_); (**D**) Precapillary resistance (R_pre_); (**E**) Postcapillary resistance (R_post_) and (**F**) Wet-to-dry ratio (W/D). (**A–E**) (○) control (n = 12), () argon (n = 13) (■) ET-1 20 nM (n = 10), () ET-1 20 nM/argon (n = 8). Statistics were performed by a LMM (**A–E**) or by the Mann-Whitney U test (**F**). *p < 0.05, **p < 0.001 and ***p < 0.001.

**Figure 2:**
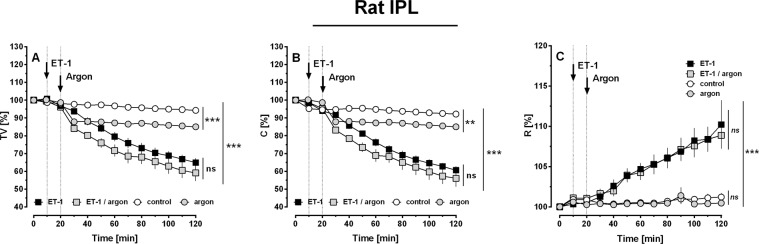
Influence of argon on airway parameters in the IPL of the rat. (**A**) Tidal volume (TV); (**B**) Lung compliance (**C**) and (**C**) Airway resistance (R). (**A–C**) (○) control (n = 12), () argon (n = 13), (■) ET-1 20 nM (n = 10), () ET-1 20 nM/argon (n = 8). (**A–C**) Statistics were performed by a LMM. **p < 0.01 and ***p < 0.001.

In IPLs, ventilation with argon was started, if baseline parameters were stable for 20 minutes. Control lungs were forward ventilated with room-air. Ventilation with argon did not alter pulmonary haemodynamics (Fig. 1A–E), e.g. pulmonary arterial pressure (P_PA_), PVR, P_cap_, R_pre_, R_post_ or oedema formation, indicated by the wet-to-dry ratio (W/D-ratio; Fig. 1F). However, ventilation with argon reduced the tidal volume (TV) (Fig. 2A; p < 0.001) and the lung compliance (C) (Fig. 2B; p < 0.01), but had no effect on the lung resistance (R) (Fig. 2C).

To study the effects of argon under conditions of increased PVR and to mimic a feature of PH^38–40^, ET-1 was added to the perfusion buffer (final concentration: 20 nM) as soon as baseline values were stable for 10 minutes. Again 10 minutes later, argon-ventilation was started. Aside from the contractile effects of ET-1 on the pulmonary vascular bed, ET-1 provokes bronchoconstriction^38^.

ET-1 significantly increased P_PA_, PVR, P_cap_, R_pre_ and R_post_ (Fig. 1A–E; all: p < 0.001), as well as the W/D-ratio (Fig. 1F; p < 0.05). Beyond that, ET-1 decreased TV and C (Fig. 2A/B; both: p < 0.01) and increased R (Fig. 2C; p < 0.001). Ventilation with argon did not alter the increasing effects of ET-1 on P_PA_, PVR and R_pre_ (Fig. 1A/B and D), but it significantly attenuated the raising effects of ET-1 on P_cap_, R_post_ and W/D-ratio (Fig. 1C/E/F; all: p < 0.05). In addition, ventilation with argon did not alter the broncho-pulmonary effects of ET-1 on TV, C or R (Fig. 2A–C).

### The effects of argon on ET-1-induced lung oedema and vascular permeability

**Figure 3:**
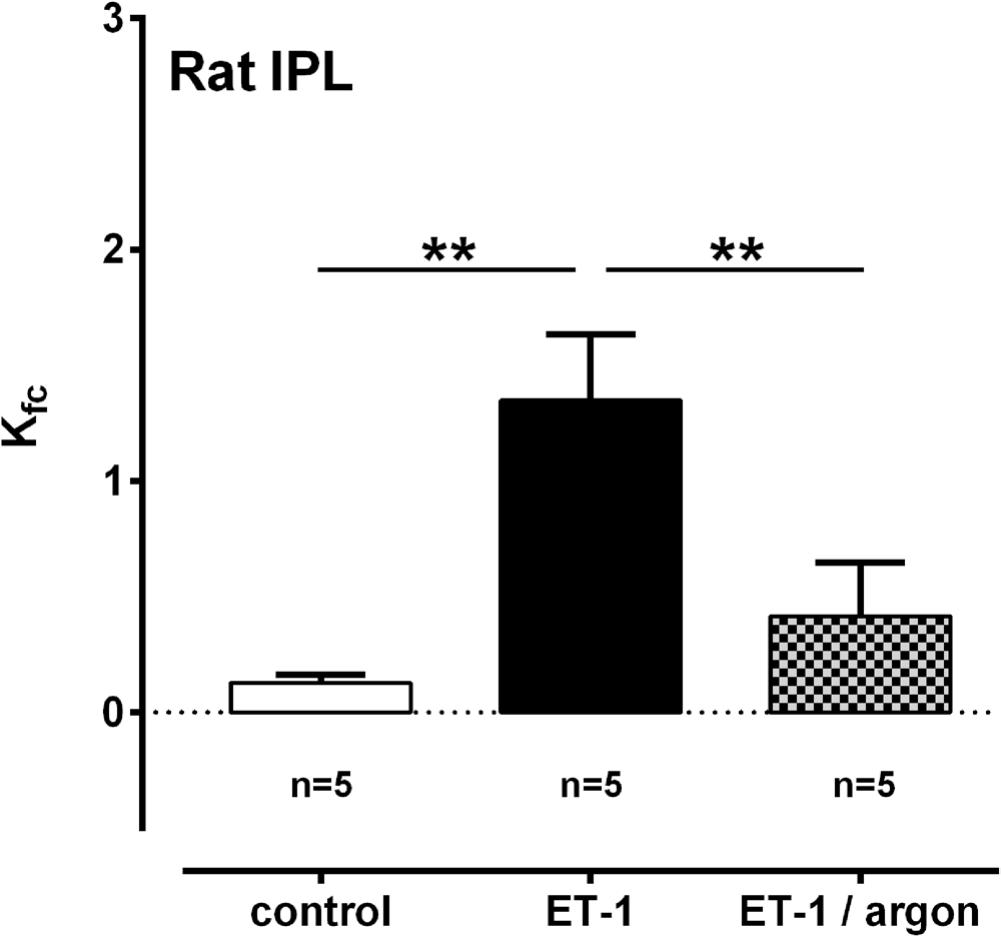
Effect of ET-1 and argon on the pulmonary filtration coefficient (K_fc_). Statistics were performed by the Mann-Whitney U test. **p < 0.001.

Argon affected the formation of ET-1-induced lung oedema (Fig. 1F). To distinguish if this effect derives from reduced P_cap_ (Fig. 1C) or from reduced vascular permeability, we perfused some lungs in addition and determined the filtration coefficient (K_fc_). Perfusion with ET-1 increased K_fc_ (p < 0.01; Fig. 3) and this effect was highly attenuated, if lungs were ventilated with an argon-mixture (p < 0.01, Fig. 3).

### The effects of argon in PCLS: Role of GABA-receptor inhibition

**Figure 4:**
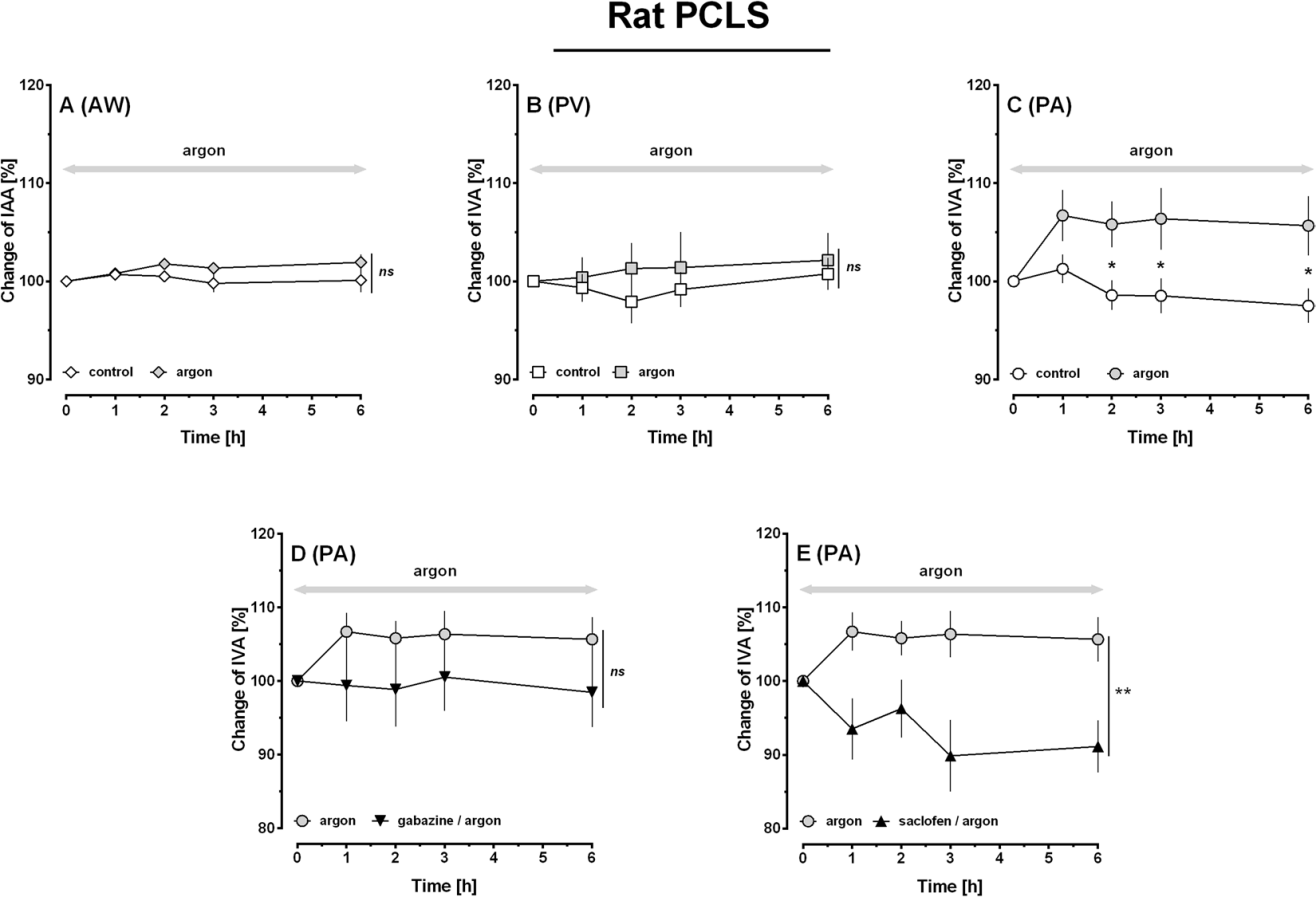
Effects of argon in PCLS of rats: Role of GABA-receptor inhibition. (**A**) Naïve AW: (◊) control (n = 15), () argon (n = 13). (**B**) Naïve PV: (□) control (n = 12), () argon (n = 13). (**C)** Naïve PA: (○) control (n = 13), () argon (n = 14). (**D**) Influence of GABA_A_-receptor inhibition on the relaxant effect of argon in PAs: () argon (n = 14), (▼) argon/gabazine (n = 5). (**E**) Influence of GABA_B_-receptor inhibition on the relaxant effect of argon in PAs: () argon (n = 14), (▲) argon/saclofen (n = 6). (**A–E**) Statistics were performed by a LMM. *p < 0.05 and **p < 0.001.

PCLS were gassed with argon in an incubation chamber; subsequently the effects of argon on the tone of airways (AWs), PVs and PAs were analysed (Fig. 4A–C). Argon did not alter the tone of AWs (Fig. 4A) or PVs (Fig. 4B). However, argon relaxed PAs, indicated by an increase of the initial vessel area (IVA) between the time points 2 h and 6 h (Fig. 4C, p < 0.05).

Next, we studied in PAs, if activation of GABA-receptors plays a role within argon-induced relaxation. Thus, PCLS were treated with the GABA_A_-receptor inhibitor gabazine or the GABA_B_-receptor inhibitor saclofen prior to exposure to argon. Control PCLS only underwent argon-gassing. Regarding IVA, the gabazine/argon group did not differ from the argon group (Fig. 4D), although a trend towards gabazine-induced inhibition of argon-induced relaxation was observable. In contrast, GABA_B_-inhibition interacted with the relaxant effect of argon (Fig. 4E; p < 0.01) and PAs even contracted slightly.

### Interaction of argon and ET-1 in PCLS: Role of GABA-receptor inhibition

**Figure 5:**
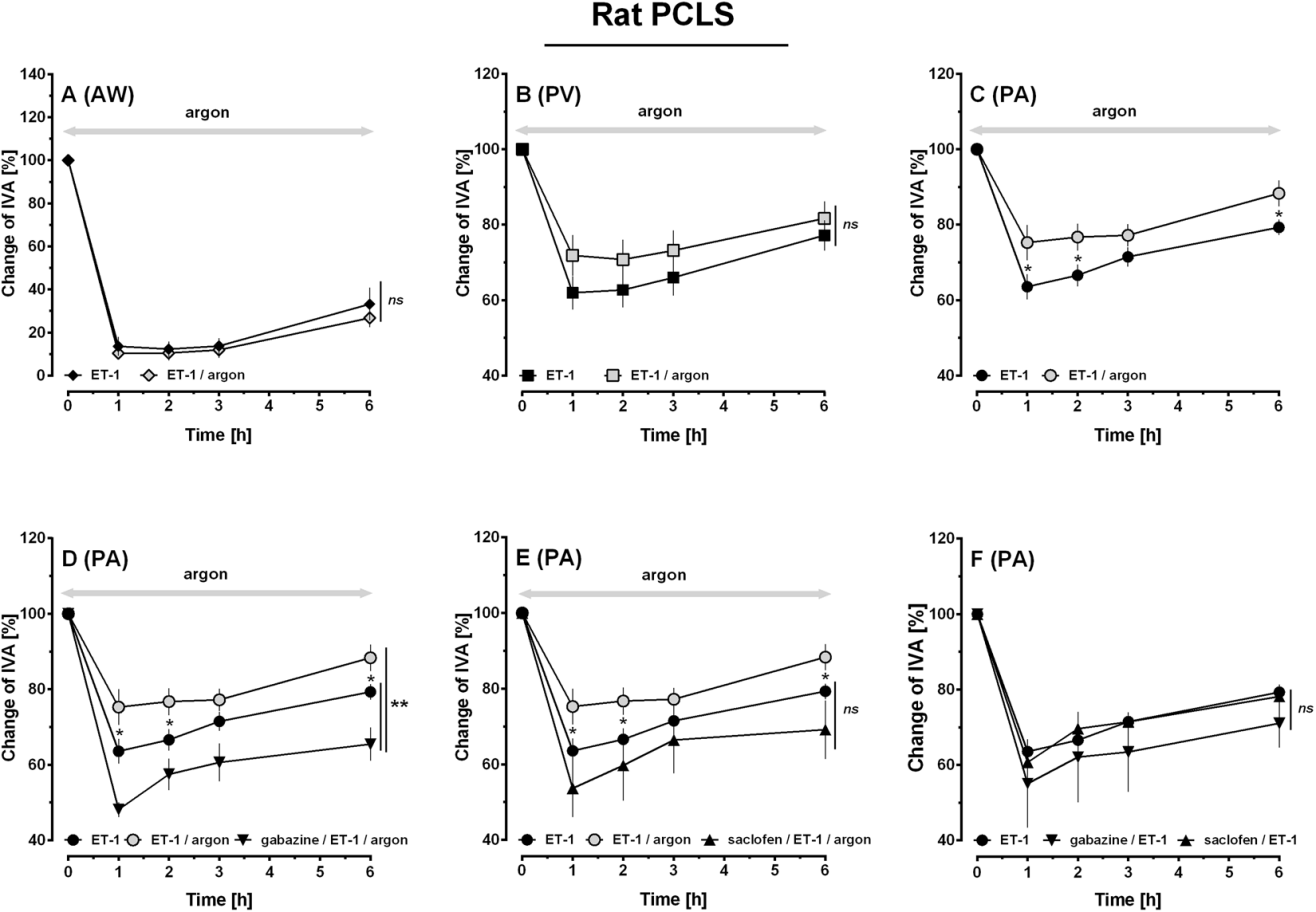
Interaction of argon and ET-1 in PCLS of rats: Role of GABA-receptor inhibition. (**A**) AW: (◆) ET-1 200 nM (n = 13), () ET-1 200 nM/argon (n = 12); (**B**) PV: (■) ET-1 200 nM (n = 14), () ET-1 200 nM/argon (n = 13); (**C)** PA: (●) ET-1 200 nM (n = 15), () ET-1 200 nM/argon (n = 13). (**D)** PA: (●) ET-1 200 nM (n = 15), () ET-1 200 nM/argon (n = 13), (▼) ET-1 200 nM/argon/gabazine (n = 4). (**E)** PA: (●) ET-1 200 nM (n = 15), () ET-1 200 nM/argon (n = 13), (▲) ET-1 200 nM/argon/saclofen (n = 5). (**F)** PA: (●) ET-1 200 nM (n = 15), (▼) ET-1 200 nM/gabazine (n = 5), (▲) ET-1 200 nM/saclofen (n = 4). (**A–F**) Statistics were performed by a LMM. *p < 0.05, **p < 0.001 and ***p < 0.001.

ET-1 contracted AWs to 13% of IAA (Fig. 5A) and PVs or PAs to 61% or 63% of IVA, respectively (Fig. 5B/C). Simultaneous gassing with argon reduced the contractile effect of ET-1 in PAs at time points 1 h, 2 h and 6 h (Fig. 5C; p < 0.05), but it did not alter the effect of ET-1 in PVs (Fig. 5B) or AWs (Fig. 5A). We wanted to highlight if activation of GABA-receptors contributes to the effect of argon within ET-1-induced contraction of PAs, thus, we pre-treated PAs with gabazine and ET-1 (Fig. 5D) or with saclofen and ET-1 (Fig. 5E) prior to their exposure to argon. Inhibition of GABA_A_-receptors (gabazine) prevented the effect of argon on ET-1-induced contraction of PAs (Fig. 5D; p < 0.05) and even increased it (Fig. 5D; p < 0.01). Inhibition of GABA_B_-receptors (saclofen) also prevented the effect of argon on ET-1-induced contraction (Fig. 5E; p < 0.05), but did not increase it (Fig. 5E; p > 0.05). In contrast, if PCLS were not exposed to argon, inhibition of GABA_A/B_-receptors did not affect the tone of naïve PAs (data not shown) and did not alter the contractile effect of ET-1 in PAs (Fig. 5F).

### Modulation of ET-1-induced contraction in PCLS: Role of GABA-receptor activation

**Figure 6:**
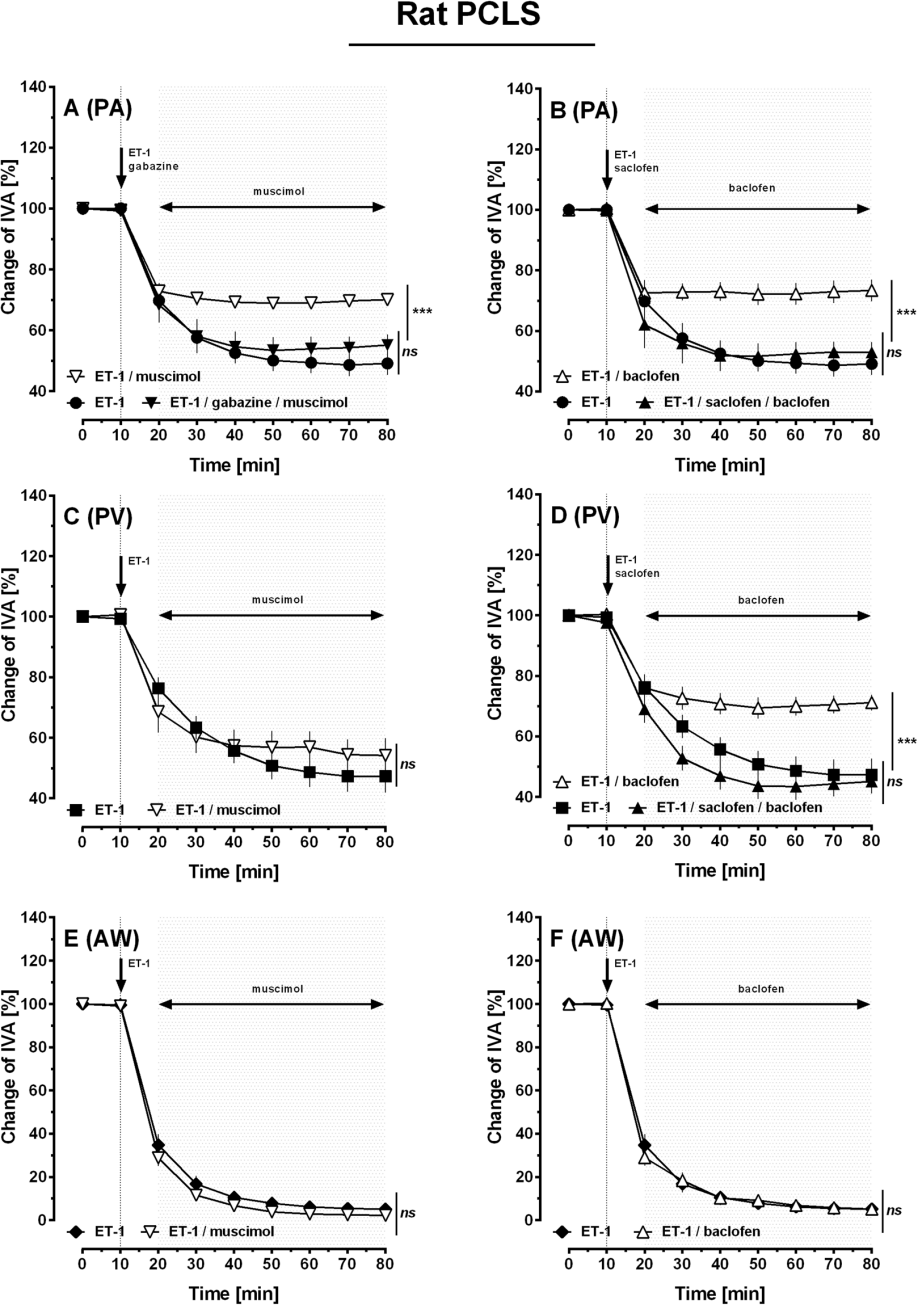
Modulation of ET-1-induced contraction in PCLS of rats: Role of GABA-receptor activation. (**A**) PA: (●) ET-1 200 nM (n = 15), (∇) ET-1 200 nM/muscimol 5 nM (n = 8), (▼) ET-1 200 nM/muscimol 5 nM/gabazine 5 µM (n = 5). (**B)** PA: (●) ET-1 200 nM (n = 15), (Δ) ET-1 200 nM/baclofen 5 nM (n = 7), (▲) ET-1 200 nM/baclofen 5 µM/saclofen 5 µM (n = 5). (**C)** PV: (■) ET-1 200 nM (n = 11), (∇) ET-1 200 nM/muscimol 5 nM (n = 7). (**D)** PV: (■) ET-1 200 nM (n = 11), (Δ) ET-1 200 nM/baclofen 5 nM (n = 8), (▲) ET-1 200 nM/baclofen 5 µM/saclofen 5 µM (n = 3). (**E)** AW: (♦) ET-1 200 nM (n = 15), (∇) ET-1 200 nM/muscimol 5 nM (n = 8). **F)** AW: (♦) ET-1 200 nM (n = 15), (Δ) ET-1 200 nM/baclofen 5 nM (n = 7). (**A–F**) Statistics were performed by a LMM. ***p < 0.001.

Inhibition of GABA_A/B_-receptors did not modulate the contractile effect of ET-1 in PAs (Fig. 5F). Next, we studied, if activation of GABA_A/B_-receptors alters ET-1-induced contraction in PAs, PVs or airways. Thus, PCLS were treated with ET-1 alone, ET-1/gabazine or ET-1/saclofen prior to the treatment with the GABA_A_-receptor agonist muscimol or the GABA_B_-receptor agonist baclofen (R/S baclofen). In PAs, muscimol decreased the contractile effect of ET-1 compared to their exposure only to ET-1 (Fig. 6A; p < 0.001). The effect of muscimol was prevented, if GABA_A_-receptors were blocked by gabazine (Fig. 6A; p < 0.001). Accordingly, exposure to the GABA_B_-receptor agonist baclofen reduced the contractile effect of ET-1 in PAs (Fig. 6B; p < 0.0001). This effect was prevented, if GABA_B_-receptors were blocked by saclofen (Fig. 6B; p < 0.001). In PVs, muscimol did not attenuate ET-1-induced contraction (Fig. 6C; p > 0.05), but baclofen reduced it (Fig. 6D; p < 0.001) which was also prevented by saclofen (Fig. 6D). In addition, muscimol or baclofen did not alter ET-1-induced bronchoconstriction (Fig. 6E/F).

### Effect of argon in untreated and ET-1 pre-treated human pulmonary arteries (PCLS)

**Figure 7:**
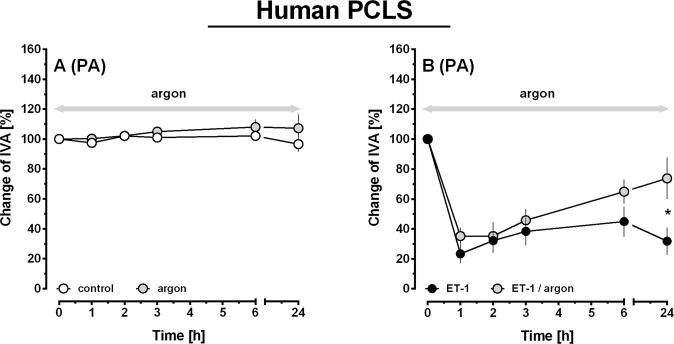
Effect of argon in untreated and ET-1 pre-treated human pulmonary arteries (PCLS). (**A)** PA: (○) control (n = 4), () argon (n = 5). **B)** PA: (●) ET-1 100 nM (n = 4), () ET-1 100 nM/argon (n = 5). (**A**,**B**) Statistics were performed by a LMM. *p < 0.05 and ***p < 0.001.

Human PAs were gassed with argon and analysed subsequently. Argon did not change the tone of naïve human PAs (Fig. 7A). ET-1 (100 nM) contracted PAs to 23% of IVA (Fig. 7B; p < 0.001) and simultaneous exposure to argon did not alter the initial contractile effect of ET-1 (Fig. 6B). However, if PAs were exposed for 24 h to argon, the contractile effect of ET-1 was attenuated (Fig. 7B; p < 0.05).

## Discussion

In this study, argon relaxed the pulmonary circulation. Gassing with an argon-mixture (argon 74%, CO_2_ 5%, O_2_ 21%) reduced the tone of rat PAs and lowered ET-1-induced contraction in PAs from rats or humans. In IPLs, ventilation with an argon-mixture reduced the ET-1-induced increase of P_cap_, R_post_ and the W/D-ratio. Regarding argon-induced relaxation, GABA-receptors appear to be involved, as 1) inhibition of GABA_B_-receptors prevented the relaxant effect of argon in naïve PAs and 2) inhibition of GABA_A/B_-receptors blocked the attenuating effect of argon on ET-1-induced contraction. Beyond that, GABA_A/B_ seems to interact with ET-1, as stimulation of GABA_A/B_- or GABA_B_-receptors reduced ET-1-induced contraction in rat PAs or PVs, respectively. In the IPL, argon exerted some effects on the airway tone which were not confirmed in PCLS and discussed later.

In rat PAs, argon exerted relaxation and reduced the contractile effect of ET-1. Both effects were evident, if argon-gassing was performed for 2 hours, whereas argon did not alter the tone of PVs, emphasizing the different response of PAs or PVs to various stimuli^30,32,41^. In line with our results from rats, argon attenuated the contractile effect of ET-1 in human PAs, although a longer duration of argon-gassing was necessary, but it did not relax naïve human PAs. The differential behaviour of PAs from both species might be due to several reasons. (1) ET-1-induced contraction differed among PAs of rats or humans and was about 63% or 25% of IVA, respectively. This fact could explain the delayed effect of argon on ET-1-induced contraction in human PAs. (2) Rat PAs relaxed without pre-contraction suggesting a certain resting tone, as it was shown for PVs from guinea pigs^30,31^. (3) Human PAs only relaxed due to argon, if they were pre-contracted, as it was shown for milrinone^31^ and other relaxant stimuli (our own unpublished data). Most likely, they do not dispose of a resting tone, 4) PAs from both species belong to different parts of the pulmonary arterial bed. PAs from rats derive from a central part of the lung, whereas human PAs derive from a more peripheral part of the lung. Regarding the diverging responses to argon, we can expect species-dependent differences^31,36^, but also that various pulmonary vascular segments react differently to similar stimuli^42^.

Despite the intriguing effects of argon on the pulmonary arterial tone of rats or humans, argon did not influence P_PA_ or R_pre_, regardless if PVR was increased by ET-1 or not. In the IPL, P_PA_ represents the central part of the pulmonary arterial bed which corresponds to rat PAs studied in PCLS, whereas R_pre_ rather displays peripheral PAs. So, it was somewhat unexpected that ventilation with argon did not affect P_PA_ in untreated lungs or did not lower the effect of ET-1 on P_PA_. Regarding these discrepancies, the following ideas should be considered. When PCLS are exposed to argon the argon-mixture reaches the PAs, PVs or AWs directly, thus allowing the study of argon’s effects with confidence. Unlike PCLS, inhaled argon initially must pass the pulmonary vasculature at the capillary and postcapillary level where, argon effectively relaxed the tissue targets. However, it is uncertain, if argon acts in other parts of the pulmonary circulation which are more distant to the alveolo-capillary membrane to the same extent, or whether higher concentrations are required, e.g. realised by an extended application period. Potentially, the pulmonary arterial relaxant effects of argon shown in PCLS are less relevant for clinical practice. Irrespective of these considerations, argon did not deteriorate pulmonary haemodynamics, as it was shown for xenon^43,44^. It rather exerted beneficial effects on P_cap_, R_post_ and oedema formation which are not debatable and of interest in patients with LHD often suffering from postcapillary PH and/or lung oedema^45^.

In the IPL, argon reduced the ET-1-induced increase of P_cap_ and prevented the effects of ET-1 on the W/D-ratio and on K_fc_. Apart from that, argon reduced the ET-1-induced increase of R_post_ resembling the postcapillary vascular bed, and thus the smallest PVs. Our data from PCLS (rats) do not reflect these results, as argon did not relax PVs and did not attenuate the contractile effect of ET-1 in PVs. Nonetheless, they become more distinct, if some other factors are considered. (1) R_post_ is determined by the tone of the smallest PVs which are not reflected in PCLS, as PCLS from rats allow the study of central, large PVs, whereas human PCLS enable the study of more peripheral, but not the smallest PVs. (2) P_LA_ reflects the central pulmonary venous system. However, if constant flow is applied during negative pressure ventilation, it is essential to establish a pressure balancing chamber in the perfusion outflow to prevent negative pressure lung oedema. This pressure balancing chamber is connected by tubes to the artificial thorax chamber. Hence, P_LA_ conforms to the pressure in the thorax chamber. Finally, we studied in PCLS central or medium-sized PVs, whereas in the IPL, the smallest parts of the pulmonary venous bed were addressed.

Here, argon lowered the ET-induced increase of R_post_, but did not influence PVR. At first look this appears to be a discrepancy, but it is explainable. PVR is determined as followed: PVR = (P_PA_ − P_LA_) × 80/flow. Due to the facts that (1) argon had no effect on P_PA_, (2) P_LA_ was fixed due to the pressure balancing chamber and (3) the flow was constant, PVR could not change. In contrast, R_post_ is calculated as followed: R_post_ = (P_cap_ − P_LA_)/flow. Based on the facts that argon lowered the ET-1-induced increase of P_cap_, whereas P_LA_ and the flow remained constant, R_post_ unavoidably decreased. We hypothesise that argon would have decreased the effect of ET-1 on PVR, if we had applied positive pressure ventilation.

Other studies addressing the pulmonary vascular effects of argon are scarce. Martens *et al*.^27^ studied the organoprotective effects of argon in a porcine model of *ex vivo* lung perfusion. PVR was increased by a warm ischaemic period of 2 hours. Afterwards, argon ventilation was started, but without effects on PVR. In a similar work, Martens *et al*.^28^ studied the organoprotective effects of argon within the context of pre-conditioning. Again, argon did not alter PVR. The studies from Martens *et al*.^27,28^ and our study show diverging results and are difficult to compare. (1) Martens *et al*. increased PVR by a warm ischaemic period, whereas we used ET-1. (2) They used a model without fixed P_LA_, but in our set-up P_LA_ was fixed. (3) They did not determine segmental PVR (R_pre_, R_post_) and (4) both studies were done in different species.

In this study, PVR was increased by ET-1 to mimic a characteristic of PH. Notably, increased ET-1-levels play a role in sepsis, sepsis-related organ dysfunction^46–48^ and within the pathogenesis of acute lung injury^46,49^. This issue is emphasised by the fact that ET-1-antagonists attenuate the occurrence of acute lung injury^50–52^. Here, argon ventilation completely prevented the effect of ET-1 on the W/D-ratio and on K_fc_. Thus, argon prevented the formation of lung oedema by a decrease of P_cap_ which is the main pressure driving fluid from the pulmonary capillaries to the interstitium^53^ and by decreased vascular permeability. This topic is clinical relevant, as patients suffering to neurological illness often develop neurogenic lung oedema being crucial for their prognosis^54^. Our results are in conflict to those of Martens *et al*.^27,28^ who did not observe argon-related effects on the W/D-ratio. Possibly, the diverging results rely on the various modes of induction of lung oedema. These conflicting results suggest that argon should be further explored within acute lung injury or lung oedema.

Argon-induced downstream signalling is unexplored. In view of neuroprotection, a role of ERK1/2, PI3K-AKT^3,19,20^, TLR2/4^17,21,22^ or Bcl-2^18^ is discussed, though all these targets are fairly unspecific. The anaesthetic effect of argon is referred to the activation of GABA_A_-receptors^24^. GABA (γ-aminobutyric acid) is the main inhibitory neurotransmitter in the mammalian brain^55^. Beyond that, GABA-receptors are found in the lungs^56,57^. There, activation of GABA_A_-receptors leads to airway smooth muscle relaxation^58–62^ and plays a role in the fetal development of the lung^63,64^.

Here, inhibition of GABA_A/B_-receptors did not affect the tone of naïve PAs and did not alter the contractile effect of ET-1 in PAs. Thus, the basal activation of GABA_A/B_-receptors appears to be not relevant. However, inhibition of GABA_B_-receptors (saclofen) reduced the relaxant effect of argon in rat PAs indicating a certain role of GABA_B_-receptors within argon-induced relaxation. The relevance of GABA for the regulation of the pulmonary vascular tone is supported by Starke *et al*.^65^ who proved in PAs from rabbits that their contractile force is reduced, if PAs were treated with GABA and further, that GABA_A_-inhibition by bicuculline or picrotoxin did not prevent this effect. Hence, a dominant role of GABA_B_ appears to be possible. Here, argon lowered the contractile effect of ET-1 in rat PAs and this effect was prevented, if GABA_A/B_-receptors were blocked by gabazine or saclofen. These data allow us to conclude that argon relaxes rat PAs via activation of GABA_A_- and GABA_B_-receptors. They are in line with data from Kaye *et al*.^66^, who found in the feline pulmonary vascular bed that both the GABA_A_-agonist muscimol and the GABA_B_-agonist SKF-97541 relaxed the pulmonary vascular bed, if it was pre-contracted with the thromboxane analogue U46619. Regarding the pulmonary vasorelaxant effects of GABA, further studies are lacking. Though, Suzuki *et al*.^67^ showed that monocrotaline-induced pulmonary vascular remodelling was attenuated, if rats were pre-treated with GABA leading to decreased levels of norepinephrine. To the best of our knowledge, the presence of GABA-receptors has been proven in the lung^56,57^, but not specifically in the pulmonary circulation, even if the activity of GABA-transaminase was verified in PAs or PVs of guinea pigs, with dominance for PAs suggesting the presence of GABA-receptors^68^. In addition, there is evidence that stimulation of GABA-receptors alters the tone of systemic vessels^69,70^.

Beyond the pulmonary vasorelaxant effects of argon, argon reduced the effects of ET-1 on the formation of lung oedema and on K_fc_. Within this context, the role of GABA is supported by several studies indicating its protective effect on the development of lung oedema, e.g. Chintagari *et al*.^71^ reported that intratracheal instillation of GABA attenuated the effect of high-tidal volume ventilation on the formation of lung oedema by an increased alveolar fluid clearance. Conversely, this effect was prevented if GABA was instilled together with the GABA_A_-antagonist bicuculline^71^. However, there is also evidence that activation of GABA_A_-receptors aggravate lung oedema^64^. These contrasting results might be explained by a switch of the Cl^−^ conductance pattern of GABA_A_-receptors according to the intracellular Cl^−^ concentration^63^. In addition, Zhang and colleagues^72^ showed that propofol also acting on GABA_A_-receptors^73^ reduces the occurrence of neurogenic pulmonary oedema.

In the IPL, we found a significant reduction in TV and C with the administration of the argon-mixture. These changes should not be related to the different viscosity of both gases, as (1) we performed a viscosity based correction of our data (explained in the method section) and (2), if we did not correct the data, TV and C would have been rather increased due to the higher viscosity of the argon-mixture. Our results from PCLS do not show any changes of the airway tone due to the exposure to the argon-mixture. They are further in line with others^12,27–29^ who did not find altered lung mechanics or blood gas analyses due to inhalation of argon. Hence, it must be questioned which phenomena might account for the noticed reduction of TV and C?

The ventilation of the IPL is initiated by a negative pressure in the lung chamber. This amounts to a pressure controlled ventilation mode, such that the TV normally should be the same (based on the lung compliance) independent of the viscosity of the argon-mixture in spite of the change in airway resistance, though the time to filling could increase. However, due to the physiologically realistic breathing frequency of 70 per minute, the increased time to fill might have caused the small difference in TV that was observed.

Though, regarding the role of GABA_A/B_-receptors within the pulmonary vasorelaxant effect of argon, it is somewhat unexpected that argon did not exert bronchorelaxation, as activation of GABA_A_-receptors was shown to relax airway smooth muscle in several studies^58–62^. In view of the pulmonary circulation, it seems that argon-mediated activation of GABA_B_-receptors is more dominant than stimulation of GABA_A_-receptors. Anyhow, if argon stimulates GABA_A_-receptors in PAs, we assume that this should be also the case in AWs. Possibly, the lack of bronchorelaxant effects of argon relies on the intensity of ET-1-induced bronchoconstriction. Obviously, ET-1 contracted PAs and PVs to 60–65% of IVA, whereas the AWs contracted to 10–15% of IAA. Most probably, bronchoconstriction was too strong for a later relaxation.

In rat PAs, activation of GABA_A/B_-receptors (muscimol/baclofen) reduced the contractile effect of ET-1. In contrast, in PVs this was only the case, if GABA_B_-receptors were stimulated. Conversely, muscimol and baclofen did not alter ET-1-induced contraction, if GABA-receptors were blocked with gabazine or saclofen, emphasising the specific activation of GABA_A/B_. In this view, it must be questioned if ET-1 acts at all on GABA_A/B_-receptors, or rather if activation of GABA_A/B_-receptors alters ET-1-induced contraction. From the literature there is some evidence that GABA interacts anyhow with ET-1, e.g. it was reported^74^ that the application of the GABA_A_-antagonist bicuculline led to the generation of lung oedema. In that study, ET-1 levels were increased in the bronchoalveolar lavage of bicuculline-treated rats^74^ and conversely, the occurrence of lung oedema was attenuated by phosphoramidon or by the ET_A_-antagonist BQ-123^74^. In addition, GABA reduces the release of norepinephrine^65,67^ which highly contributes to the formation of neurogenic lung oedema^75,76^.

In conclusion, argon decreased the pulmonary vascular tone of the rat, if PVR was enhanced, but it did not affect the airway tone. In view of the pulmonary vasorelaxant potential of argon, activation of GABA_A/B_-receptors plays a pivotal role. Finally, our results support the application of argon for neuroprotection in patients with critical pulmonary haemodynamics based on PH, RV failure or LHD. The relevance of our findings is strengthened by the fact that argon also relaxed human PAs.

## Methods

### Animals and human lung tissue

Female Wistar rats (250 ± 50 g) were purchased from Charles River (Sulzfeld, Germany) and used as lung donors. Rat lungs were randomly assigned to one of the groups.

Human PCLS were prepared from patients undergoing lobectomy due to cancer. After pathological inspection, cancer free tissue from a peripheral pulmonary part was used. None of the patients showed any signs of PH (echocardiographic or histological evaluation). The study was approved by the local ethics committee (EK 61/09) of the Medical Faculty Aachen, Rhenish-Westphalian Technical University Aachen. All patients gave written informed consent.

All animal studies and experimental procedures were approved by the Landesamt für Natur, Umwelt und Verbraucherschutz North Rhine-Westphalia (ID: 84-02.04.2013.A146; ID: 8.87-51.05.20.10.245; ID: 50086A4) and performed due to the Directive 2010/63/EU of the European Parliament.

### IPL: Preparation

IPLs were prepared as described before^31,35,77^. Briefly, female rats were anaesthetised with 95 mgkg^−1^ pentobarbital (Narcoren; Garbsen, Germany) and bled, if reflex checks were unresponsive. The trachea was cannulated and the lungs were ventilated with positive pressure (70 breath/min). In addition, a PEEP of 3 cmH_2_O and an I:E of 1:1 were applied. As soon, as cannulas were inserted into the pulmonary artery (inflow) and the left atrium (outflow), the lungs were perfused at constant flow (20 ml/min) with 200 ml Krebs-Henseleit buffer, containing 2% bovine serum albumin, 0.1% glucose, 0.3% HEPES and 50 nM salbutamol to prevent bronchoconstriction^78^. The buffers’ temperature was kept at 37 °C by a water bath and the pH was maintained between 7.35 and 7.45 by gassing with CO_2_. After pulmonary ventilation and perfusion was established, heart and lungs were removed and set into a negative-pressure chamber which was adjusted for P_min_ = −7 cmH_2_O and P_max_ = −2 cmH_2_O. To prevent lung oedema during constant flow perfusion and negative pressure ventilation, a pressure balancing chamber was set in the perfusion outflow and connected by tubes to the artificial thorax chamber. To prevent atelectasis, every 5 minutes a deep breath was applied. Airway (TV, C or R) and pulmonary haemodynamic parameters (P_PA_, P_LA_ and flow) were recorded with the Pulmodyn Software 2.0 (Hugo-Sachs Elektronik Harvard Apparatus, Germany). If all parameters were stable, the PVR was enhanced with ET-1 (final buffer concentration: 20 nM)^79^.

### IPL: Calculation of airway parameters

The pressure inside the thorax chamber was measured by a pressure transducer (Hugo-Sachs Elektronik Harvard Apparatus, Germany). The inhalation flow (Q) was measured by a pneumatograph (Hugo-Sachs Elektronik Harvard Apparatus, Germany). Finally, TV was calculated from the integration of Q. The compliance (C) of the lung expresses its elasticity. C is defined by the TV and the change (Δ) of the transpulmonary pressure (P_tp_). In the IPL, ΔP_tp_ reflects the difference of the maximal and minimal pressure (P) in the thorax chamber. So, C was calculated: C = TV/(P_max_ − P_min_) and R (resistance) was calculated by the inhalation flow (Q) in relation to P_max_ and P_min_: R = (P_max_ − P_min_)/Q^35^.

### IPL: Ventilation with argon and correction of airway parameters

To ventilate the lungs with argon, a pressure regulator and a flow meter were used. Argon (flow 0.3 L/min) was applied via a tube which was connected to the pneumatograph. Inhalation flow was measured using a pneumatograph consisting of a single tube with small enough diameter to maintain laminar flow such that the pressure drop across the tube is linearly proportional for the flow rate. The complication in this application occurs because the proportionality is dependent on the gas viscosity (***µ***); i.e., if the gas viscosity is increased the pressure drop needed to drive the flow would be increased. The pneumatograph was originally calibrated for air; thus for the argon mixture that has a greater viscosity (2.227 kg/(s-m)10^−5^ for argon at 32 °C versus 1.84 kg/(s-m)10^−5^ for air at 32 °C) the measured pressure drop will be greater than that for air at the same flow rate. Therefore, a recalibration of the pneumatograph for the argon-mixture would be necessary; however this is not applicable without interrupting the experiment. Another possibility is to correct the pressure drop data by multiplying by the viscosity ratio of air over argon to obtain the flow rate. Alternatively, TV, R and C can be corrected. We corrected TV, R and C as followed: TV_corr_ = TV_meas_ × (***µ***_air_/***µ***_argon-mix_); R_corr_ = R_meas_ × (*** µ***_air_/***µ***_argon-mix_) and C_corr_ = TV_corr_/(P_max_ − P_min_)^80^.

### IPL: Calculation of PVR, precapillary and postcapillary resistance

PVR was calculated as followed: ${\rm{PVR}}=({{\rm{P}}}_{{\rm{PA}}}-{{\rm{P}}}_{{\rm{LA}}})\ast 80/{\rm{flow}}$. To determine R_pre_ and R_post_, P_cap_ was recorded every 10 minutes by the double occlusion method. R_pre_ and R_post_ were calculated according to the following equation: ${{\rm{R}}}_{{\rm{pre}}}=({{\rm{P}}}_{{\rm{PA}}}-{{\rm{P}}}_{{\rm{cap}}})/{\rm{flow}}$ and ${{\rm{R}}}_{{\rm{post}}}=({{\rm{P}}}_{{\rm{cap}}}-{{\rm{P}}}_{{\rm{LA}}})/{\rm{flow}}$^27,81^.

### IPL: Wet-to-dry ratio (W/D-ratio)

After IPLs were perfused for 2 h, the wet weight of the right superior lobe was recorded and subjected to drying at 60 °C for 72 h. The dry weights were monitored and the W/D-ratio was calculated.

### IPL: Assessment of the vascular permeability by determination of the filtration coefficient

To distinguish, if lung oedema derives from increased P_cap_ or increased vascular permeability, the capillary filtration coefficient (K_fc_) was determined as described in reference^35^. Measurements were performed at 0 and 120 minutes of the perfusion using the following equation: K_fc_ = (dweight/dtime)/dP_cap_. Due to the fact, that weight gain measurements do not allow the simultaneous application of the double occlusion method, P_cap_ was calculated according to the Gaar equation^82^: P_cap_ = P_LA_ + 0.44 (P_PA_ − P_LA_).

### PCLS of rats and humans: Preparation

Rats received intraperitoneal anaesthesia with pentobarbital, which was verified by missing reflexes. Thereafter, they were prepared as described before^31,36^. Rat lungs were filled via the trachea and human lungs were filled via a main or lobar bronchus, respectively, with 1.5% low-melting agarose. Afterwards, they were cooled on ice. Tissue cores (diameter 11 mm) were prepared and cut into about 250 µm thick slices with a Krumdieck tissue slicer (Alabama Research & Development, Munford, USA). PCLS were incubated over night at 37 °C and repeated medium changes were performed to wash out the agarose.

### PCLS: Treatment and videomicroscopy

To study the role of GABA within argon-induced relaxation, PCLS were treated with the GABA_A_-receptor antagonist gabazine (5 µM)^83^ or with the GABA_B_-receptor antagonist saclofen (5 µM)^84^ (Fig. 4D/E). To study the relaxant effect of argon in pre-contracted PAs, PVs and AWs, rat PCLS were pre-contracted with 200 nM ET-1 (Fig. 5A–C) and human PCLS were pre-contracted with 100 nM ET-1 (Fig. 7B). To study the role of GABA-receptors within argon-induced relaxation in ET-1 pre-contracted PAs, PCLS were simultaneously pre-treated with 200 nM ET-1 and 5 µM gabazine (Fig. 5D) or with 200 nM ET-1 and 5 µM saclofen (Fig. 5E). To study the effect of GABA-receptor inhibition or activation within ET-1 induced vasoconstriction and bronchoconstriction, PCLS were pre-treated with ET-1 and gabazine (Fig. 6A), ET-1 and saclofen (Fig. 6B/D) or ET-1 alone (Fig. 6C/E/F) prior to the treatment with the GABA_A_-receptor agonist muscimol (5 nM)^85,86^ or the GABA_B_-receptor agonist baclofen (5 µM)^87^. In PCLS, all changes of the IVA and IAA were quantified in % and indicated as “Change of IVA [%]” or “Change of IAA [%]”. Thus, an IVA < 100% indicates contraction and an IVA > 100% indicates relaxation. In order to compare the effect of argon in pre-treated vessels, the intraluminal area was defined after pre-treatment again as 100%. In the graphs, all pre-treatments were indicated. The intraluminal area of PAs, PVs and airways was monitored with a digital video camera (Leica Viscam 1280, Leica DFC 280). The images were analysed with Optimas 6.5 (Media Cybernetics, Bothell, WA).

### PCLS from rats and humans: Argon gassing

PCLS were transferred into 24 well plates (1 ml medium per well). In order to treat PCLS with argon (Argon 74%, CO_2_ 5%, O_2_ 21%) or air mix (N_2_ 74%, CO_2_ 5%, O_2_ 21%), we used the modular incubator chamber (Billups-Rothenberg, USA) with a filling volume of 6 liters. To avoid evaporation, we added 20 ml of purified water in a petri dish inside the chamber. Next, the incubator chamber with the PCLS inside was purged with the appropriate gas mixture and a gas flow rate of 20 L/min for 5 minutes; the outlet and inlet ports where closed with plastic clamps. Alkalisation of the incubation medium was prevented, as all gas mixtures were enriched with 5% CO_2_. Afterwards, the whole chamber was transferred in a heat CO_2_ incubator. In order to investigate the effects of argon on pre-constricted airways or vessels, we pre-treated PCLS with ET-1 and made images at 1 h, 2 h, 3 h, 6 h and even 24 h, in case of human PCLS. To study the role of GABA within the relaxant effect of argon, we pretreated PCLS with the GABA inhibitors gabazine or saclofen and made images according to the pre-treatment with ET-1. Images were recorded by videomicroscopy and the IVA/IAA was calculated with Optimas 6.5.

### Reagents

ET-1 was purchased from Biotrends (Wangen, Switzerland). The potency of ET-1 differs strongly with age and lot numbers. GABA receptor agonists/antagonists and standard laboratory chemicals were from Sigma-Aldrich (Steinheim, Germany). Gas mixtures were delivered from Air Liquide GmbH (Simmerath, Germany) or Linde Gas AG (Pullach, Germany).

### Statistical analysis

Statistics was conducted using SAS 9.2 (SAS Institute, Cary, North Carolina, USA) and GraphPad Prism 5.01 (GraphPad, La Jolla, USA). All data were analysed by a linear mixed model analysis, except Figs 1F and 3 which were analysed by the Mann Whitney U Test. P-values were adjusted for multiple comparisons (false discovery rate) and presented as mean ± SEM. N indicates the number of animals or lung lobes. P < 0.05 are considered as significant: *p < 0.05, **p < 0.01, ***for p < 0.001.

### Ethical approval and informed consent

Animal studies were approved by the Landesamt für Natur, Umwelt und Verbraucherschutz North Rhine-Westphalia (ID: 84-02.04.2013.A146; ID: 8.87-51.05.20.10.245; ID: 50086A4) and performed due to the Directive 2010/63/EU of the European Parliament.

Human PCLS were prepared from patients undergoing lobectomy due to cancer. All patients gave written informed consent and the local ethics committee (EK 61/09) of the Medical Faculty Aachen, Rhenish-Westphalian Technical University Aachen, approved the study.

## Data Availability

The datasets generated and analysed during the current study are available from the corresponding author on reasonable request.
